# Distribution of Primary and Specialized Metabolites in *Nigella sativa* Seeds, a Spice with Vast Traditional and Historical Uses

**DOI:** 10.3390/molecules170910159

**Published:** 2012-08-24

**Authors:** Ilan Botnick, Wentao Xue, Einat Bar, Mwafaq Ibdah, Amnon Schwartz, Daniel M. Joel, Efraim Lev, Aaron Fait, Efraim Lewinsohn

**Affiliations:** 1 Department of Vegetable Crops, Newe Ya’ar Research Center, Agricultural Research Organization, P.O. Box 1021, Ramat Yishay 30095, Israel; Email: butnik@gmail.com (I.B.); bareinat@volcani.agri.gov.il (E.B.); mwafaq@volcani.agri.gov.il (M.I.); 2 The Institute of Plant Sciences and Genetics in Agriculture, Faculty of Agricultural, Food and Environmental Quality Sciences, The Hebrew University of Jerusalem, P.O. Box 12, Rehovot 76100, Israel; Email: schwartz@agri.huji.ac.il; 3 The Jacob Blaustein Institutes for Desert Research, Ben-Gurion University of the Negev, Beer-Sheva 84105, Israel; Email: xwt19861103@gmail.com (W.X.); fait@bgu.ac.il (A.F.); 4 Deptartment of Phytopathology and Weed Research, Newe Ya’ar Research Center, Agricultural Research Organization, P.O. Box 1021, Ramat Yishay 30095, Israel; Email: dmjoel@volcani.agri.gov.il; 5 Department of Erets Israel Studies, University of Haifa, Haifa 31905, Israel; Email: elev@univ.haifa.ac.il

**Keywords:** black cumin, *Nigella sativa*, Ranunculaceae, thymoquinone, *p*-cymene, monoterpenes, nigellidine, nigellicine, Cairo’s Genizah

## Abstract

Black cumin (*Nigella sativa* L., Ranunculaceae) is an annual herb commonly used in the Middle East, India and nowadays gaining worldwide acceptance. Historical and traditional uses are extensively documented in ancient texts and historical documents. Black cumin seeds and oil are commonly used as a traditional tonic and remedy for many ailments as well as in confectionery and bakery. Little is known however about the mechanisms that allow the accumulation and localization of its active components in the seed. Chemical and anatomical evidence indicates the presence of active compounds in seed coats. Seed volatiles consist largely of olefinic and oxygenated monoterpenes, mainly *p-*cymene, thymohydroquinone, thymoquinone, γ-terpinene and α-thujene, with lower levels of sesquiterpenes, mainly longifolene. Monoterpene composition changes during seed maturation. γ-Terpinene and α-thujene are the major monoterpenes accumulated in immature seeds, and the former is gradually replaced by *p*-cymene, carvacrol, thymo-hydroquinone and thymoquinone upon seed development. These compounds, as well as the indazole alkaloids nigellidine and nigellicine, are almost exclusively accumulated in the seed coat. In contrast, organic and amino acids are primarily accumulated in the inner seed tissues. Sugars and sugar alcohols, as well as the amino alkaloid dopamine and the saponin α-hederin accumulate both in the seed coats and the inner seed tissues at different ratios. Chemical analyses shed light to the ample traditional and historical uses of this plant.

## 1. Introduction

*Nigella sativa* L. seed (also called black cumin, or black seed) is an annual herb of the Ranunculaceae family. Black cumin is native to southern Europe, North Africa, south and southwest Asia and has been traditionally used since ancient times as an important medicinal plant and spice [1]. *N. sativa* is one of the most ancient known domesticated plants and its seeds were reportedly found in Tutankhamon’s tomb [2]. Black cumin is referred by its Hebrew name “Ketzah” in the Bible in the book of Isaiah 28:25-27 and known for its curative properties. Black cumin seeds have been used as condiment and for medicine in many cultures along history [3]. In ancient Babylonia the plant was used externally to treat swelling, the hair, and bruises, and internally to cure stomach problems [4]. Classical physicians such as Hippocrates and Galen described the use of black cumin to treat various maladies, including infections in the nose, while Dioscorides described the plant and its black seeds with their pungent smell, and reports their use as food and for curative purposes to treat headaches and toothache, to cure diseases of the eyes and skin and leprosy, to eliminate intestinal worms, to accelerate menstruation, to increase urine flow and milk flow, and to repel snakes [1,5]. The physician Assaf sets out medical uses, for example, to treat colds in the head, chest and body, to kill intestinal worms, to increase semen and increase virility; to cure leprosy, bright skin spots, infections in the nose, and to enrich hair growth. It also served as a component in a medication against poisons and the stings of venomous creatures [6,7].

Muslim medicine regarded black cumin seeds as a medicine for colds and many other diseases [8,9]. For example, the holy *Sahih al-Bukhari* book mentions black cumin as “a cure for every disease except death” [10]. Avicenna referred to black seed in his *Canon of Medicine* as the seed that stimulates the body’s energy and helps recovery from fatigue and dispiritedness [11]. al-Kindī describes the use of the seeds in a preparation against skin irritations and in a medication against insanity [4,12]. Ibn al-Baytar cites al-Tamimi, who relates the use of black cumin oil against paralysis and facial spasms [13]. al-Qazwini cites various physicians who describe the use of the plant to eliminate fleas and mosquitoes, to remove face freckles, to straighten the hair, to expel crawling insects, to remove skin moles, and to treat leukodermia albinum, leprosy, colds, and toothache [14,15]. 

**Figure 1:**
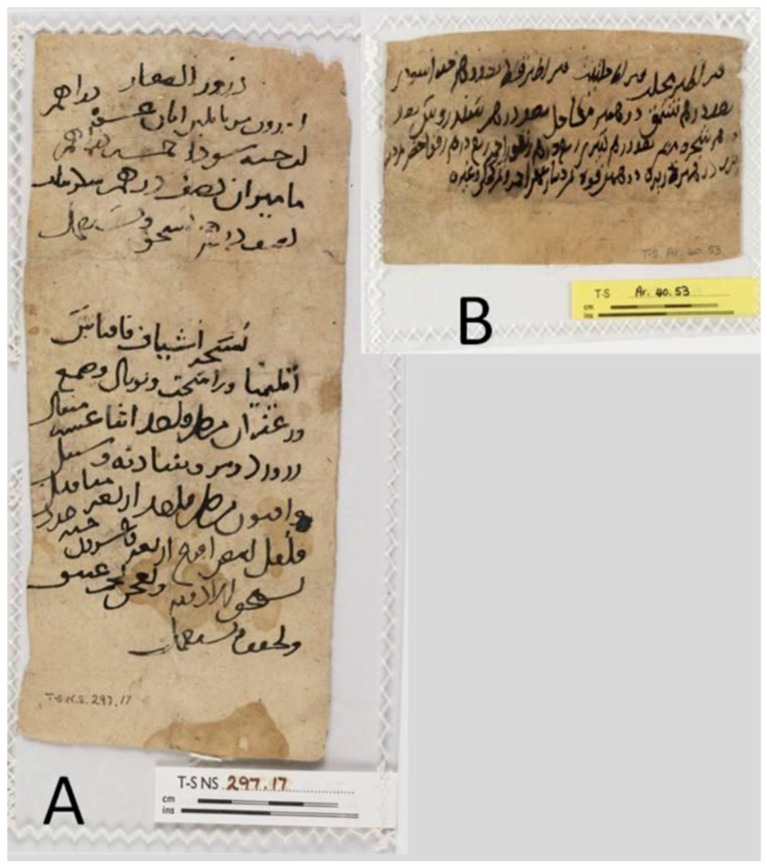
Medieval practical medical prescriptions, found in the Cairo Genizah. The documents are written in Arabic on paper (**A**) Exhibit T-S NS 297.17 showing two practical medical prescriptions. The first (top) prescription mentions among other substances seeds of black cumin (3rd row); the second prescription is for the preparation of an eye powder using myrrh, opium, saffron and various minerals; (**B**) Another practical medical prescription, probably for the treatment of hemorrhoids (Exhibit T-S Ar.40.53) mentioning seeds of black cumin.

Maimonides notes the use of cumin to prepare a sneezing powder, to reduce facial swellings, to prepare a medication against bites, and to treat the bite of a poisonous spider [7,16]. Black cumin figures twice in a list of *materia medica* dating from the middle ages, found in the Genizah of Cairo’s old synagogue [17]; and in two prescriptions as an emmenagogue and as an abortifacient (Figure 1). It is also mentioned in medical books on ophthalmology, paediatrics, fevers, and poisons. In present day traditional medicine, ripe *N. sativa* seeds are used to treat many ailments such as respiratory difficulties, hepatic and digestive disorders, diarrhea, cold, heartache and inflammatory disorders, conjunctivitis, chest congestion, asthma, flatulence and polio [1,10,11,18,19,20]. The seeds are considered a general immunostimulant and therefore the fixed oil extraction of black cumin is traditionally taken orally as a tonic on a daily basis [20]. Traditionally, mature seeds are employed and used in infusions, pomades or by inhalation. Black cumin seeds are also commonly used as a spice and added to pastries, dairy products and salads*.*

The vast therapeutic characteristics of *N. sativa* seeds are due to their unique phytochemical composition [1,19,21]. The seeds contain about 35 to 41% fixed oil, mainly composed by the non-saturated linoleic, oleic and palmitic acids. Seeds also contain 11,14-*cis*,*cis*-eicosadienoic acid (a unique ω-6 fatty acid) [22,23]. The volatile oil of the seed (0.5–1.6%) is composed mainly of the monoterpenes *p*-cymene, γ-terpinene, α-pinene, β-pinene, α-thujene, carvacrol and thymoquinone [24,25]. Thymoquinone is considered the major active compound of *N. sativa* [1]. Thymoquinone has antioxidant activity [25], and has been shown to induce apoptosis and adversely affect cell division in cancer cells [11,26,27]. Thymoquinone blocked angiogenesis *in vivo*, and inhibited the growth of prostate and colon tumors implanted in nude mice with no noticeable side effects. Overall, results indicate that thymoquinone effectively inhibits tumor angiogenesis and tumor growth and could be used as a promising potential drug candidate for cancer therapy [28]*.* Indazole alkaloids such as nigellicine and nigellidine, as well as the isoquinoline alkaloids nigellimine and nigellimine *N*-oxide, together with dolabellane-type diterpene alkaloids have been isolated from seeds [19,29,30,31,32,33]. The saponin α-hederin, that has pharmacoactive properties and is also present in common ivy (*Hedera helix* L.), is also a constituent of *N. sativa* seeds [34,35].

The unique volatile oil of *Nigella sativa* seeds imparts its special aroma and contributes to its taste. In spite of the culinary and accepted pharmacological importance of *N. sativa*, little is known about the mechanisms that allow the accumulation and localization of its active components in the seed. Here we describe changes in the volatile oil composition of *N. sativa* seed during seed development and the distribution of primary and specialized metabolites in *N. sativa* seed.

## 2. Results and Discussion

### 2.1. Volatile Oil Characterization in Mature N. sativa Seeds

The volatile composition of *N. sativa* mature seeds is shown in Table 1. Twenty five compounds were identified in *N. sativa* seeds from both sources with no significant differences between the two. The major fraction (90% w/w) of the volatile oil of the mature seeds consisted of monoterpenes and the remaining compounds were mainly sesquiterpenes, being longifolene the most abundant one present. Monoterpenes present in mature seeds included *p*-cymene, thymohydroquinone, α-thujene, thymoquinone, γ-terpinene and carvacrol. Other components included α-pinene, β-pinene and *trans* 4-methoxythujane. These compositions are reportedly similar in seeds cultivated in other parts of the world such as Austria, India, Algeria and Poland [24,25,36,37]. Interestingly, the volatiles of *N. sativa* seed resemble qualitatively the compositions of other monoterpene-phenol-rich Lamiaceae aromatic plants, such as *Thymus*, *Satureja* and *Origanum* [38,39,40], although the levels of thymoquinone in these Lamiaceae spp. are very low (less than a few %) as compared to *N. sativa* (Table 1).

### 2.2. Changes in the N. sativa Seed Volatiles during Seed Maturation

**Figure 2:**
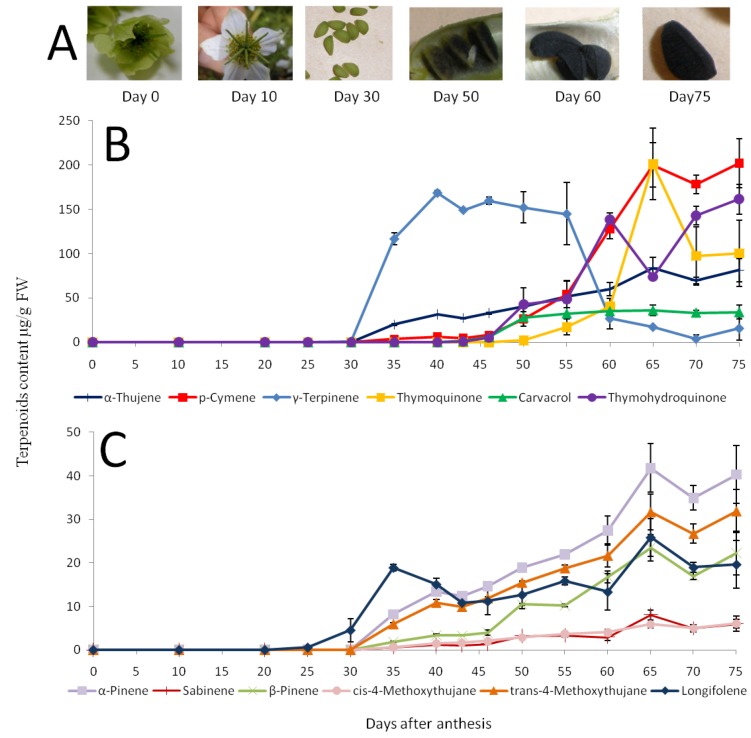
Changes in volatile levels during *N. sativa* seed maturation. (**A**) Developmental stages of *N. sativa* seed. (**B**) Major components. (**C**) Minor components. Means and SE of three replicates of samples from Ein Harod source are shown.

The ripening process of *N. sativa* seeds can be divided into six characteristic stages (Figure 2A): During the first stage-anthesis-the petaloid sepals are yellow and flowering starts (day 0). In the second stage–the flower is fully open and the petaloid sepals are white in color (day 10). 

**Table 1:** Volatiles of mature *Nigella sativa* seeds from two seed sources. The volatiles were extracted with MTBE and identified by GC-MS as described in Materials and Methods. Means are average of 3 replicates ± SE.

Seed source			Ein-Harod			Naan	
Compound			µg/g F.W.	% (W/W)	µg/g F.W.		% (W/W)
Monoterpene hydrocarbons	I.M. *	R.I.					
α-thujene	MS,RI,AS	925	43.8 ± 6.8	9.7 ± 0.3	51.7 ± 11.4		10.4 ± 0.3
α-pinene	MS,RI,AS	940	21.9 ± 4.2	4.8 ± 0.2	24.3 ± 4.5		4.9 ± 0.2
sabinene	MS,RI,AS	970	3.9 ± 0.7	0.9 ± 0.04	4.7 ± 1.1		1 ± 0.04
β-pinene	MS,RI,AS	975	11 ± 2	2.4 ± 0.1	12.7 ± 2.8		2.6 ± 0.1
myrcene	MS,RI,AS	985	2.1 ± 0.2	0.5 ± 0.1	1.9 ± 0.5		0.4 ± 0.04
α-terpinene	MS,RI,AS	1014	5.5 ± 1.8	1.2 ± 0.4	1.5 ± 0.3		0.3 ± 0.1
*p*-cymene	MS,RI,AS	1024	113 ± 17.3	25 ± 0.5	128 ± 25.9		25.9 ± 0.02
limonene	MS,RI,AS	1030	3.1 ± 0.6	0.7 ± 0.03	3.1 ± 0.6		0.6 ± 0.04
γ-terpinene	MS,RI,AS	1055	52.1 ± 22.4	11.6 ± 5.5	4.5 ± 0.2		0.9 ± 0.2
terpinolene	MS,RI,AS	1086	1.3 ± 0.1	0.3 ± 0.1	1 ± 0.2		0.2 ± 0.02
total			257	57.1	233.33		47.3
**Monoterpene alcohols**							
terpinene 4-ol	MS,RI,AS	1181	2 ± 0.4	0.4 ± 0.01	2.6 ± 0.7		0.5 ± 0.1
carvacrol	MS,RI,AS	1298	26.1 ± 3.9	5.8 ± 1	16.9 ± 4.1		3.4 ± 0.9
thymohydroquinone	MS,RI	1550	73.0 ± 15.7	16.2 ± 1.9	114.5 ± 15.1		23.2 ± 2.4
total			101.1	22.5	134		27.1
**Monoterpene ethers**							
*cis*-4-methoxythujane	MS,RI	1094	3 ± 0.6	0.7 ± 0.04	3.5 ± 0.9		0.7 ± 0.04
*trans*-4-methoxythujane	MS,RI	1118	17 ± 3	3.8 ± 0.2	19.9 ± 4.6		4 ± 0.1
4,5-epoxy-1-isopropyl-4-methyl-1-cyclohexene	MS	1201	4.1 ± 0.9	0.9 ± 0.1	4.7 ± 1.5		0.9 ± 0.1
total			24.1	5.4	28.2		5.7
**Monoterpene ketones**							
carvone	MS,RI,AS	1245	0.2 ± 0.1	0.04 ± 0.01	0.3 ± 0.1		0.1
thymoquinone	MS,RI,AS	1253	35.2 ± 17.5	7.8 ± 3.7	67.7 ± 23.7		13.7 ± 2.3
total			35.4	7.9	68		13.8
**Monoterpene ester**							
bornyl acetate	MS,RI,AS	1286	0.4 ± 0.1	0.1 ± 0.01	0.5 ± 0.1		0.1 ± 0.01
total			0.4	0.1	0.5		0.1
**Aldehydes**							
2 *E*,4*Z*- decadienal	MS,RI	1295	0.3 ± 0.02	0.1 ± 0.01	0.3 ± 0.03		0.1 ± 0.01
2 *E,*4*E-* decadienal	MS,RI	1319	0.5 ± 0.1	0.1 ± 0.01	0.7 ± 0.1		0.1 ± 0.01
total			0.8	0.2	0.9		0.2
**Sesquiterpenes**							
longipinene	MS,RI	1355	3.4 ± 0.5	0.8 ± 0.04	2.7 ± 1.3		0.5 ± 0.2
longifolene	MS,RI	1415	17.1 ± 1.8	3.8 ± 0.20.04 ± 0.01	13.3 ± 5.8		2.7 ± 1
*trans-*caryophyllene	MS,RI,AS	1423	0.2 ± 0.1		0.2 ± 0.03		0.03
zonarene	MS,RI	1523	0.6 ± 0.2	0.1 ± 0.04	0.6 ± 0.1		0.1
total			21.3	4.7	16.7		3.4
**Unidentified**			14.1 ± 3.4	2.4	16.7 ± 5.3		2.3
**Total essential oil**			450.3 ± 69.5	100	493.7 ± 99.5		100

* I.M.: Identification method: comparison with: MS: Mass spectrum from computerized library; RI: Retention Index; AS: Authentic standard.

The third stage—as a result of fertilization—the seeds are apparent and a green seed coat is clear, developing inside a green follicle fruit (day 30). During the fourth stage–black spots appear on the seed coat (day 50). During the fifth stage the seed coat is entirely black in color and partially hardens (day 60). Finally during the sixth “mature” stage (day 75) the ripe seed is fully swollen and hard.

Interestingly, monoterpene levels and their composition changed dramatically during seed maturation. Extracts from flowers contained mainly 2*E*-hexanal, 2*E*-hexenol and the sesquiterpene *t*-caryophyllene (not shown). Except of this latter compound, there was practically no accumulation of any of the characteristic compounds of the *N. sativa* mature seed volatile oil (Table 1) in flowers (not shown) or in immature seeds before 30 days after anthesis (DAA, Figure 2). The accumulation of the major volatiles (more than 5% of the total volatile fraction of the mature seed) during seed development is shown in Figure 2B. The first monoterpene detected in developing seeds was γ-terpinene, that was first discernible at 30 DAA and dramatically increasing up to a level of 170 µg/g FW by 40 DAA (Figure 2B). Then, after 55 DAA there was a marked decrease in the level of γ-terpinene reaching 30 µg/g FW at 60 DAA and its level continued to decrease till the end of seed maturation to 15 µg/g FW (Figure 2B). In addition to γ-terpinene accumulation, there was a lower constant accumulation trend of α-thujene during seed maturation, reaching 80 µg/g FW at 65 DAA (Figure 2B). Other components showing a constant accumulation trend during seed maturation included α-pinene, β-pinene, *trans*-4-methoxythujane, *cis*-4-methoxythujane and sabinene, but their levels were minor (Figure 2C). Interestingly, the accumulation of *p*-cymene starts markedly after γ-terpinene accumulation, at 55 DAA, together with the observed decreases in γ-terpinene levels (Figure 2B). *p*-Cymene is the major component of mature *N. sativa* seed volatile oil (Table 1, Figure 2B, 75 DAA). Thymohydroquinone and carvacrol also display constant accumulation trends starting at 50 DAA and peaking upon seed maturation. Thymoquinone apparently starts to accumulate 5 days thereafter, being discernible at 50 DAA, reaching its maximal levels at 65 (Figure 2B), and decreasing again towards the end of seed maturation (Figure 2B). The sesquiterpene longifolene follows a steady accumulation pattern being apparent at 30 DAA reaching its maximal level at the completion of the seed maturation process (Figure 2C). Similar trends were observed when examining developing "Na'an" seeds (not shown).

**Figure 3:**
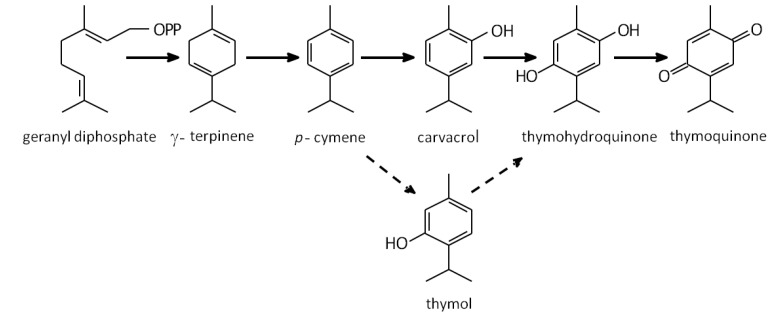
Proposed biosynthetic pathway to thymoquinone in *N. sativa* seed. The pathway is based on the better-studied pathways in the Lamiaceae [40,41] and the accumulation of the putative precursors in developing *N*. *sativa* seed (Figure 2). Geranyl diphosphate is probably cyclized to γ-terpinene, aromatized into *p*-cymene and then followed by hydroxylations to carvacrol and thymohydroquinone and oxidation to thymoquinone (upper solid arrows). In species and chemotypes accumulating thymol instead of carvacrol, hydroxylation to thymol as an alternative biosynthetic pathway is proposed (broken arrows).

Like other aromatic plants, the volatile oil of *N. sativa* is mainly composed of olefinic and oxygenated monoterpenes. The volatile oils of oregano (*Origanum vulgare* L.) and thyme (*Thymus vulgaris* L., both Lamiaceae) bear a strong resemblance to the composition of *N. sativa* volatiles (Table 1) but they are accumulated in glandular trichomes present on leaf surfaces [40]. Moreover, γ-terpinene is a precursor of *p*-cymene in both thyme and oregano [40,41]. Based on their findings we hypothesize that γ-terpinene may also serve as a precursor of *p*-cymene in *N. sativa* seed (Figure 2). This assumption is supported by the observed strong decrease in γ-terpinene levels at 55 DAA in parallel to the marked increase in *p*-cymene (Figure 2B), the major component of *N. sativa* seed. In addition, we hypothesize that *p*-cymene is a precursor of carvacrol in *N. sativa* as it is for thymol in thyme and in oregano [41,42]. Most of the *N. sativa* seeds characterized have been shown to accumulate carvacrol, but some accessions apparently accumulate thymol instead of carvarcrol [43,44] or in addition to it [24,25,36,37]. Interestingly, other plant species that accumulate carvacrol in their volatile oils also have chemotypes that accumulate thymol [38]. Considering the structural similarity between the different monoterpene components of *N. sativa* volatile oil and their presence in species that accumulate similar compounds we suggest that the biosynthetic pathway to carvacrol and thymoquinone follows a similar pattern to the much better studied pathway to thymol in the Lamiaceae (Figure 3). Our suggested model is supported by the succession in the accumulation of such compounds in developing *N. sativa* seeds.

Due to the taxonomical divergence between the Lamiaceae and Ranunculaceae we speculate that the presence of similar volatile oil compositions based mainly on phenolic monoterpene alcohols and their derivatives, as well as the seemingly similar biosynthetic pathway is another example of convergent evolution, a well documented phenomenon in plant specialized metabolism [45]. Further biochemical studies and the availability of the genes coding for the key enzymes catalyzing thymoquinone biosynthesis will contribute to our understanding of phyllogenetic origin and the metabolic pathways to phenolic monoterpene alcohols and thymoquinone in *N. sativa*.

### 2.3. Distribution of Specialized and Other Metabolites within N. sativa Seed

**Figure 4:**
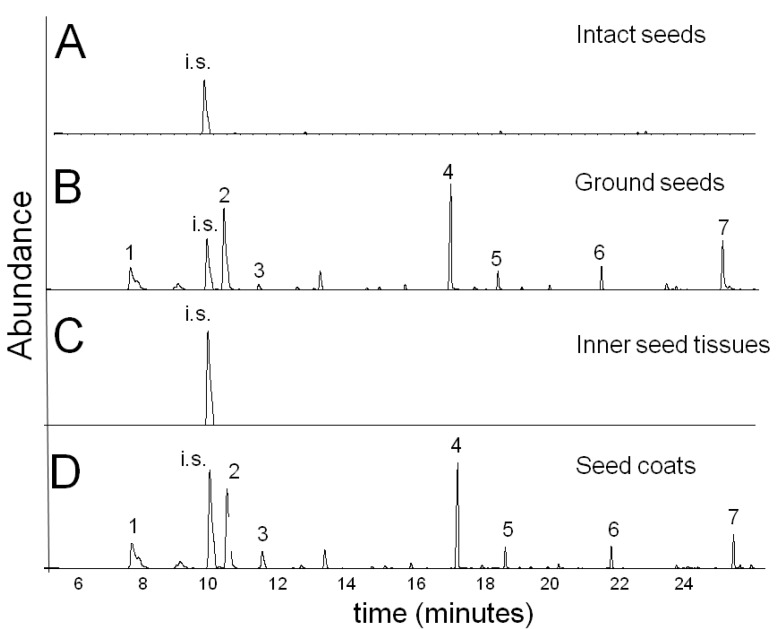
Distribution of mono-and sesquiterpene volatiles in *N. sativa* mature seed. The volatiles were solvent extracted and analyzed by GC-MS as described in Materials and Methods. **1**: α-thujene; **2**: *p*-cymene; **3**: γ-terpinene; **4**: thymoquinone; **5**: carvacrol; **6**: longifolene; **7**: thymohydroquinone; i.s. internal standard (isobutyl benzene). (**A**) Mature intact seed. (**B**) Ground complete seed. (**C**) Inner seed tissues (endosperm and embryo). (**D**) Seed coats.

To study the distribution of specialized and central metabolites between the seed coat and the seed inner parts (endosperm and embryo), the seed-coat was separated from the endosperm and the two tissues were analyzed independently. The volatile mono- and sesquiterpenes of *N*. *sativa* seeds could not be extracted from intact seeds (Figure 4A), but were readily extracted after the seed coats were crushed prior to the extraction (Figure 4B). No volatile terpenes were detected in the inner endosperm and embryo tissues (Figure 4C leaves, shoots or roots (data not shown). Still, the seed coats extracts displayed high levels of mono- and sesquiterpenes (Figure 4D). The inability to readily extract the volatile oil components from intact seeds (Figure 4A), but its facile extraction from isolated seed coats indicates that the volatile oil does not accumulate on the surface of the seed coat, but likely accumulates in inside layers. Conversely, the volatiles of okra seed (*Abelmoschus esculentus*, Malvaceae) can readily be extracted by gently shaking the whole intact seeds in hexane, indicating that in okra seed the volatiles accumulate in the outer epidermis layer [46]. 

Specialized metabolites [45] often accumulate in anatomically distinct structures such as oil ducts, cavities, idioblasts, glandular epidermis or glandular trichomes, that compartmentalize these often toxic components from other metabolically active cells [47,48,49,50]. The mericarps of many plants of the family Apiaceae such as fennel (*Foeniculum vulgare*), caraway (*Carum carvi*) and anise (*Pimpinella anisum*) are commonly referred as seed spices, although anatomically they are fruits. These tissues accumulate their volatile oil in special cavities named oil ducts [51]. We failed to observe such structures in *N. sativa* seeds. 

**Figure 5:**
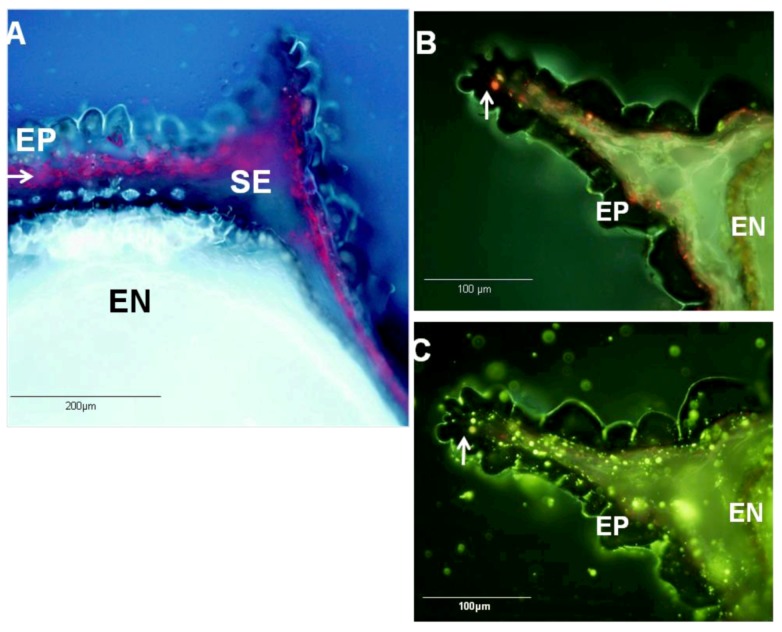
Fluorescence micrographs of longitudinal sections of mature *N. sativa* seed. (**A**) Autofluorescence under UV light excitation. Pink-fluorescent droplets are observed in the subepidermal layer (SE) of the seed coat. (**B**) Blue light excitation fluorescence. The lipophilic droplets display a red fluorescence indicated by white arrows. (**C**) The same section under blue light excitation after staining with fluorol yellow 088, the droplets display a yellow fluorescence typical to lipophilic material (white arrows). Legend: EN–Endosperm tissue; EP–Epidermal cells; SE–Subepidermal layer.

Longitudinal sections of mature *N. sativa* seeds were examined under the fluorescent microscope. Histological examination indicated that fluorescent material accumulates in the subepidermal layer of the seed coats, in the form of droplets distributed evenly throughout this layer (Figure 5A–C). The anatomy *N. sativa* and other *Nigella* spp seed was previously described [52], but the patterns of accumulation of specialized metabolites were previously unknown. The typical fluorescence of the droplets under UV and blue light excitation may be attributed to the presence of phenolic and other aromatic compounds such as thymohydroquinone and thymoquinone. Indeed, a similar fluorescence hue was typical to that of authentic thymoquinone observed under the same conditions (not shown). Using the reagent Fluorol Yellow 088, the droplets stained and fluoresced in a characteristic yellow color typical to lipophilic material (Figure 5C). Still, other lipophilic material present in the endosperm and other seed tissues and clearly not associated with the volatile oil, also fluoresced following this staining. Moreover, the dye solvent (ethanol) seems to have dispersed much of the lipophilic material, though not in intact cells. This problem rendered fluorol yellow 088 of only partial value for the specific localization of monoterpenes in *N. sativa* seed sections. Our histological results are however consistent with the assumption that the volatile oil of *N. sativa* is accumulated in the inner layers of the outer seed coat, but further experimental proof is needed to unequivocally determine the composition of the fluorescent material observed. 

**Figure 6:**
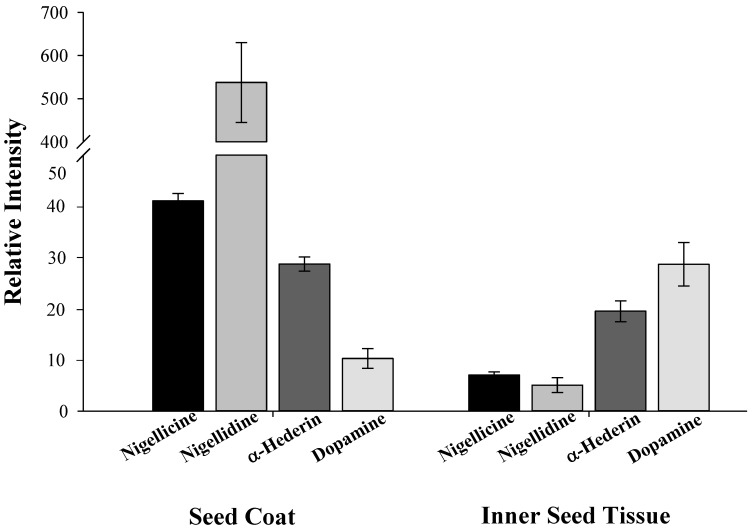
Distribution of alkaloids and α-hederin in seed coats and inner seed tissues.of *N. sativa.* Analyses were performed on a UPLC-Q-TOF-MS, except for dopamine, that was detected by GC-MS. Relative content is given as the metabolite response (metabolite peak intensity/internal standard peak intensity/FW). The error bars in graphs refer to the mean values ± SE of four replicates. In all cases significant differences were noted between the metabolite levels in seed coats as compared to the endosperm tissues (*t* test, *p* < 0.05).

Other specialized and pharmacoactive metabolites are distinctively unevenly distributed within the seed tissues of *N. sativa*. The alkaloids nigellidine and nigellicine accumulate almost exclusively in the seed coats (Figure 6). In contrast, the phenylethylamino alkaloid dopamine is present mainly in the inner seed tissues, but lower levels of dopamine are also prominent in the seed coats. Dopamine has never been reported to occur in *N. sativa* seeds, but is present in banana and is a known precursor to other alkaloids [53]. In contrast, the saponin α-hederin is present in both seed tissues at comparable levels (Figure 6). 

**Figure 7:**
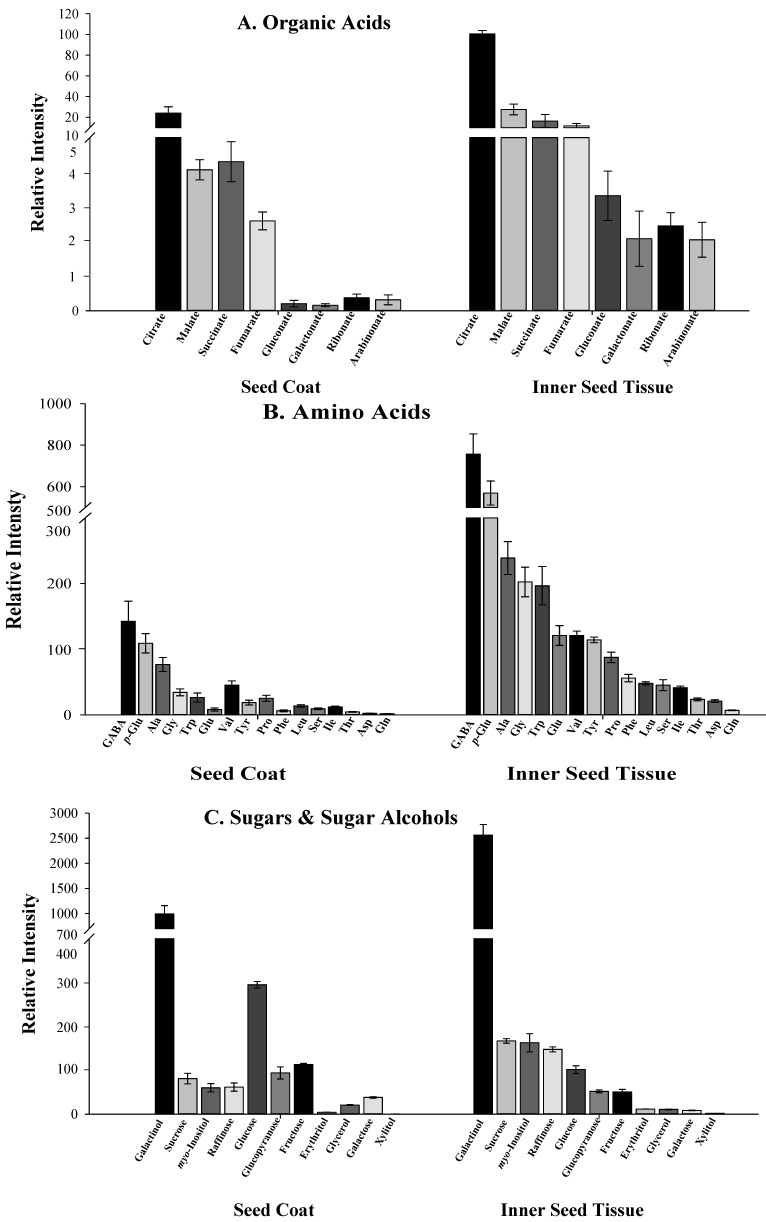
Distribution of central metabolites in theseed coat and inner seed tissues of *N. sativa.* Analyses were performed by GC-MS after derivatization with methoxyamine hydrochloride (20 mg/mL in pyridine). Relative content is given as the metabolite response (metabolite peak intensity/internal standard peak intensity/FW). The error bars in graphs refer to the mean values ± SE of four replicates. All the metabolites shown displayed significantly differences between seed coats and inner seed tissues (*t* test, *p* < 0.05). (**A**) Organic acids; (**B**) Amino acids; (**C**) Sugars and sugar alcohols.

Expectedly, intermediates of the central metabolism (including mainly amino and organic acids) were most prominently present in the inner tissues of the seed, and to a much lesser extent represented in the seed coats (Figure 7). These metabolites are involved in seed germination and seedling establishment, providing carbon and nitrogen to the reorganization of the metabolism in the embryo supporting root elongation and later seedlings establishment [54]. The absence of central metabolic processes in the seed coat suggests that this tissue bears a functional role of volatile oil storage and possible defense. The presence of dedicated transport mechanisms of precursors of volatile compounds from the inner parts of the seed to the seed coats is therefore hypothesized. That said, the distribution of the sugars and sugar alcohols within *N. sativa* seed followed a seemingly inconsistent pattern (Figure 7). Some compounds such as glucose and its cyclic isoform glucopyranose, galactose, glycerol and fructose, are present in both tissues but their levels are higher in the seed coats. These metabolites are mainly components of cell walls, or precursors of storage compounds such as starch and triacylglycerols (TAGs). Their presence in the seed coat is likely related to the presence of lipid and extensive cell wall structures in this tissue. Other compounds such as raffinose, erythritol, sucrose and myo-inositol, although present in both tissues, are higher in the inner parts of the seed. The question whether active metabolism exists in the seed coat and if the metabolites identified are involved in volatile biosynthesis is standing. 

### 2.4. Ecological Relevance to the Distribution of Metabolites within Black Cumin Seed

Although the pharmacological activity and the acceptance of *N. sativa* as a natural drug is unquestionable, the actual ecological role of the terpenoids and alkaloids in *N. sativa* seed is a matter of speculation. The accumulation of the volatile terpenes in the inside layer of the seed coat layer allows for the storage of the metabolites and their release only after a relatively strong injury, either mechanical or mediated by insects or microorganisms, as well as following significant periods of time, likely including the rinsing action of rainfall. The volatile oil of *N. sativa* has antibacterial [19] antifungal [55,56] and insecticidal [57] activities and therefore, such compounds may play an important role in keeping the seed competitive for germination. Some components present in the seed coats of *N. sativa* such as carvacrol, thymohydroquinone and thymoquinone inhibit seed germination and might be allelopathic [58,59] or might regulate *Nigella* germination. 

Although the medicinal properties of *N. sativa* have been known for centuries, we have only recently gained knowledge regarding the chemistry and the pharmacology of many of its active principles, as is the case with many commonly used spice plants and natural medicinals. Metabolomics and other genomic oriented studies will certainly enrich our knowledge on the biology of the accumulation patterns of such important plant constituents, as well as the enzymatic and genetic machinery that control their production. 

## 3. Experimental

### 3.1. Chemicals

α-Pinene, β-pinene, carvone, sabinene, *p*-cymene, γ-terpinene, carvacrol, thymoquinone and Fluorol Yellow 088 were from Fluka- Sigma-Alldrich (St. Louis, MO, USA). Bornyl acetate and terpinolene were from Carl Roth GmbH (Karlsruhe, Germany). Myrcene and α-terpinene were from SCM Chem (Phoenix, AZ, USA); *tert*-butyl methyl ether and glycerol were from Bio-lab (Jerusalem, Israel).

### 3.2. Plant Material

*N. sativa* seeds were collected from farmers in two locations in Israel: Ein-Harod (Jezre'el Valley, Lower Galilee, 32°33'37"N, 35°23"27"E) and Na'an (Sharon Plain, Central Israel, 31°52'51"N, 34°51'34"E). Voucher specimens have been deposited in the Newe Ya'ar Research Center herbarium (NSEH001, NSNAAN001 and NSLEW001. Seeds were sown in an open-air plot located at the Newe Ya'ar Research Center in Northern Israel (32°42'00"N, 35°11'00"E). Plants were irrigated and fertilized mimicking a commercial regime. Flowers were marked on day 0 when the yellow petaloids (just before anthesis) were apparent in the apical flower. The apical flowers and their fruits were then sampled every three to five days as indicated. 

### 3.3. Separation of Seed Coat from Inner Seed Tissues

Mature *N. sativa* seeds of commercial origin (200 mg) were soaked in deionised distilled water (DDW) for two to six hours. Then, the seed coat was easily separated from the inner seed tissues (containing the embryo and endosperm) with a pair of tweezers and analyzed separately for extraction of volatiles and non volatile components (see below). 

### 3.4. Extraction and Analysis of Volatile Compounds

*N. sativa* seeds (“Ein Harod”, 0.5 to 1 g) at different developmental stages and originating from three separate plants were frozen in liquid N_2_ and ground with a mortar and pestle (unless mentioned differently). Extraction of the volatile fraction was made by adding a 3 to 1 ratio (v/w) of *tert*-butyl methyl ether containing 1 ppm of isobutylbenzene as an internal standard. After a short vortex, the ground seeds were shaken for 2 h at room temperature and the extract was passed through a sodium sulfate (Merck, Darmstadt, Germany) column (Pasteur pipette) to remove excess water [60]. 

After sample concentration one μL of the extract was injected into a GC-MS (Agilent Technologies Palo Alto, CA, USA) equipped with a Restek Rtx-5 sil MS capillary column (30 m × 0.25mm × 0.25 μm). The inlet was set to 250 °C in splitless mode. Helium at a flow rate of 0.8 mL/min was used as carrier phase. The initial temperature was 50 °C and temperature gradient was programmed to 5 °C/min. till 190 °C and then 15 °C/min. to 300 °C and held for 10 min. The quadrupole detector temperature was set to 280 °C and scanning range was between 41–350 *m/z* [60]. 

### 3.5. Extraction and Analysis of Non-Volatile Compounds

Collected material was extracted and analyzed by GC-MS after derivatization using a protocol optimized for *Arabidopsis* seeds [54,61]. Relative metabolite content was calculated as described in Roessner *et al.* [62], following peak identification using TagFinder [63]. Substances were identified by comparison to mass spectral tags represented in our in-house database [64,65,66]. α-Herderin, nigellidine and nigellicine were identified in the positive ion mode by UPLC-Q-TOF-MS (Waters Corp., Manchester, UK) based analysis. A 500 μL volume of the methanol-water phase was filtered and 5 μL were injected in a UPLC-Q-TOF-MS system equipped with an ESI interface (LC: Waters Acquity UPLC system; MS: Xevo™ Q-TOF/MS (Waters Corp., Manchester, UK) operated under the following conditions. The MS conditions were as follows: Capillary voltage: +3.0 keV; Sampling cone voltage: 27 V; Extraction cone voltage 4 source temperature: 120 °C; desolvation temperature: 300 °C; cone gas flow: 50 L/h; desolvation gas flow: 650 L/h; collision energy: 6 eV; detection mode: scan (*m/z* 100–2,000; scan time 0.15 s; interscan delay: 0.05 s, centroid); dynamic range enhancement mode: off. During sample running the mobile phase consisted of 95% water: 5% acetonitrile: 0.1% formic acid (phase A), and 0.1% formic acid in acetonitrile (phase B). The solvent gradient was: 100–60% phase A over the first 8 min, 60–0% phase A over 1 min and return to the initial 100% A in 3.5 min, and conditioning for 2.5 min at 100% A. The scans were repeated for 15 min in a single run. The raw data were recorded with the aid of MassLynx version 4.1 software (Waters). Metabolites were identified by using MassLynx software and searched against the Chemspider metabolite database (http://www.chemspider.com/). The quantification of the compounds is based on the relative peak response area of each mass signal after Pareto scaling in the chromatograms and normalized to the tissue dry weight). 

### 3.6. Hystochemistry

Mature seeds were hand-cut into longitudinal sections with a razor blade. Observations were made with an OlympusX61 epifluorescence microscope (Olympus, Tokyo, Japan) using excitation UV light 360 nm or blue light 430 nm. The fluorol yellow 088 reagent stock solution [67,68] contained 0.005% (w/v) Fluorol Yellow 088 (Sigma) dissolved in 50% (v/v) PEG 400 and 45% (v/v) glycerol and DDW. The sections were stained between 1 to 10 min in a solution of the reagent diluted 1,000 fold. 

## 4. Conclusions

Black cumin is a revered medicinal plant since antiquity in Muslim and many other cultures. Monoterpene composition changes upon seed maturation, in a pattern that reflects the proposed biosynthetic pathway to thymoquinone, the major active ingredient of black cumin seeds and oil. Volatile oil components and nigellidine and nigellicine alkaloids are exclusively present in seed coats. Other compounds such as dopamine are present in the inner seed tissues, while other important metabolites are distributed in the inner seed tissues and seed coat tissues at various ratios. Black cumin is a good example of a valued and traditionally used plant that is still commonly used but whose chemical composition is little known. With the advent of modern metabolomic and genomic methodologies, we are not only beginning to understand the distribution and nature of the active principles of this seed, but also the biosynthetic pathways and their regulation. 

## References

[1] Padhye S., Banerjee S., Ahmad A., Mohammad R., Sarkar F.H. (2008). From here to eternity-the secret of Pharaohs: Therapeutic potential of black cumin seeds and beyond. Cancer Ther..

[2] Zohary D., Hopf M. (2000). Domestication of Plants in the Old World.

[3] Chevallier A.M. (1996). The Encyclopedia of Medicinal Plants.

[4] Levey M. (1966). The Medical Formulary.

[5] Gunther R.T. (1959). The Greek Herbal of Dioscorides.

[6] Muntner S.  (1967–1969). *Assaph (Harofe) the Physician* (in Herbrew). Sefer Refuoth: *Korot 4*.

[7] Muntner S. (1966). Moshe Ben Maimon (Maimonides),Treatise on Poisons and Their Antidotes (in Hebrew).

[8] Al-Akili M.M. (1993). Natural Healing with the Medicine of the Prophet: From the Book of the Provisions of the Hereafter.

[9] Levey M. (1961). Ibn Mäsawaih and His Treatise on Simple Aromatic Substances. J. Hist. Med. Allied. Sci..

[10] Butt M.S., Sultan M.T. (2010). *Nigella sativa*: Reduces the risk of various maladies. Crit. Rev. Food Sci..

[11] Zaid H., Silbermann M., Ben-Arye E., Saad B.  (2012). Greco-Arab and Islamic herbal-derived anticancer modalities: From tradition to molecular mechanisms. Evid. Based Complement. Alternat. Med..

[12] Ibn Sina (1877). Kitab al-Qanun fī al-tibb. “The Canon of Medicine”.

[13] Ibn al-Baytar (1874). Kitab al-Jami li-Mufradat al-Adwiya wa-l-Aghdhiya. “The Comprehensive Book of Simple Drugs”.

[14] al Qazwini (1981). Abu Yahya Zakariya' ibn Muhammad. Kitab Aja’ib al-makhluqat wa Gharaib al-Mawjudat, “The Wonders of Creation”.

[15] al-Antaki Dawud.  (1935). Tadhkirat uli al-albab wa-l-jami lil ’ajab al-‘ujab, “The Memorandum of the Intelligent, a Compendium of Wonders”.

[16] Rosner F., Muntner S. (1970). Moshe Ben Maimon (Maimonides), The Medical Aphorisms of Moses Maimonides.

[17] Lev E., Amar Z. (2006). Reconstruction of the inventory of *materia medica* used by members of the Jewish community of medieval Cairo according to prescriptions found in the Taylor-Schechter Genizah collection, Cambridge. J. Ethnopharmacol..

[18] Ghanzafar S.A. (1994). Handbook of Arabian Medicinal Plants.

[19] Ali B.H., Blunden G. (2003). Pharmacological and toxicological properties of *Nigella sativa*. Phytother. Res..

[20] Ramadan M.F. (2007). Nutritional value, functional properties and nutraceutical applications of black cumin (*Nigella sativa* L.): An overview. Int. J. Food Sci. Tech..

[21] Khan M.A. (1999). Chemical composition and medical properties of *Nigella sativa* Linn. Inflammopharmacology.

[22] Aitzetmuller K., Werner G., Ivanov S.A.  (1997). Seeds oils of *Nigella* species and of closely related genera. OCL-Ol. Corps Gras Lip..

[23] Houghton P.J., Zarka R., de las Heras B., Hoult J.R. (1995). Fixed oil of *Nigella sativa* and derived thymoquinone inhibit eicosanoid generation in leucocytes and membrane lipid peroxidation. Planta Med..

[24] Benkaci-Ali F., Baaliouamer A., Meklati B.Y., Chemat F. (2007). Chemical composition of seed essential oils from Algerian *Nigella sativa* extracted by microwave and hydrodistillation. Flavour Frag. J..

[25] Burits M., Bucar F. (2000). Antioxidant activity of *Nigella sativa* essential oil. Phytother. Res..

[26] Banerjee S., Padhye S., Azmi A., Wang Z., Philip P.A., Kucuk O., Sarkar F.H., Mohammad R.M. (2010). Review on molecular and therapeutic potential of thymoquinone in cancer. Nutr. Cancer.

[27] Chehl N., Chipitsyna G., Gong Q., Yeo C.J., Arafat H.A. (2009). Anti-inflammatory effects of the *Nigella sativa* seed extract, thymoquinone, in pancreatic cancer cells. HPB.

[28] Yi T., Cho S.G., Yi Z., Pang X., Rodriguez M., Wang Y., Sethi G., Aggarwal B.B., Liu M. (2008). Thymoquinone inhibits tumor angiogenesis and tumor growth through suppressing AKT and extracellular signal-regulated kinase signaling pathways. Mol. Cancer Ther..

[29] Ur-Rahman A., Malik S., Ahmed S., Chaudhry I., Ur-Rehman H. (1985). Nigellimine-*N*-oxide—A new isoquinoline alkaloid from seeds of *Nigella sativa*. Heterocycles.

[30] Ur-Rahman A., Malik S., Cun-Hung H., Clardy J. (1985). Isolation and structure determination of nigellicine, a novel alkaloid from seeds of *Nigella sativa*. Tetrahedron Lett..

[31] Ur-Rahman A., Malik S., Zaman K. (1992). Nigellimine: A new isoquinoline alkaloid from the seeds of *Nigella sativa*. J. Nat. Prod..

[32] Ur-Rahman A., Malik S., Hassan S.S., Choudhary M.I., Ni C.Z., Clardy J. (1995). Nigellidine—A new indazole alkaloid from seeds of *Nigella sativa*. Tetrahedron Lett..

[33] Morikawa T., Xu F., Kashima Y., Matsuda H., Ninomiya K., Yoshikawa M. (2004). Novel dolabellane-type diterpene alkaloids with lipid metabolism promoting activities from the seeds of *Nigella sativa*. Org. Lett..

[34] Kumara S.S.M., Huat B.T.K. (2001). Extraction, isolation and characterisation of antitumor principle, α-Hederin, from the seeds of *Nigella sati*. Planta Med..

[35] Sieben A., Prenner L., Sorkalla T., Wolf A., Jakobs D., Runkel F., Häberlein H. (2009). α-Hederin, but not hederacoside C and hederagenin from *Hedera helix*, affects the binding behavior, dynamics, and regulation of β_2_-adrenergic receptors. Biochemistry.

[36] Singh G., Marimuthu P., de Heluani C.S., Catalan C. (2005). Chemical constituents and antioxidant potentials of essential oil and acetone extract of *Nigella sativa* seeds. J. Sci. Food Agric..

[37] Wajs A., Bonikowski R., Kalemba D. (2008). Composition of essential oil from seeds of *Nigella sativa* L. cultivated in Poland. Flavour Frag. J..

[38] Skoula M., Gotsiou P., Naxakis G., Johnson C.B. (1999). A chemosystematic investigation on the mono- andsesquiterpenoids in the genus *Origanum* (Labiatae). Phytochemistry.

[39] Grosso C., Figueiredo A.C., Burillo J., Mainar A.M., Urieta J.S., Barroso J.G., Coelho J.A., Palavra A.M.F. (2009). Enrichment of the thymoquinone content in volatile oil from *Satureja montana* using supercritical fluid extraction. J. Sep. Sci..

[40] Crocoll C., Asbach J., Novak J., Gershenzon J., Degenhardt J.  (2010). Terpene synthases of oregano (*Origanum vulgare* L.) and their roles in the pathway and regulation of terpene biosynthesis. Plant Mol. Biol..

[41] Grosso C., Figueiredo A.C., Burillo J., Mainar A.M., Urieta J.S., Barroso J.G., Coelho J.A., Palavra A.M.F. (2010). Composition and antioxidant activity of *Thymus vulgaris* volatiles: Comparison between supercritical fluid extraction and hydrodistillation. J. Sep. Sci..

[42] Poulose A.J., Croteau R. (1978). Biosynthesis of aromatic monoterpenes conversion of γ-terpinene to *p*-cymene and thymol in *Thymus vulgaris*. Arch. Biochem. Biophys..

[43] D’Antuono L.F., Morreti A., Lovato A.F.S. (2002). Seed yield, yield components, oil content and essential oil content and composition of *Nigella sativa* L. and *Nigella damascena* L. Ind. Crops Prod..

[44] Hamrouni-Sellami I., Kchouk M.E., Marzouk B.  (2008). Lipid and aroma composition of black cumin (*Nigella sativa* L.) seeds from Tunisia. J. Food Biochem..

[45] Pichersky E., Lewinsohn E. (2011). Convergent evolution in plant specialized metabolism. Annu. Rev. Plant Biol..

[46] Camciuc M., Bessiere J.M., Vilarem G., Gaset A. (1998). Volatile components in okra seed coat. Phytochemistry.

[47] Caissard J.C., Joly C., Bergougnoux V., Hugueney P., Mauriat M., Baudino S. (2004). Secretion mechanisms of volatile organic compounds in specialized cells of aromatic plants. Rec. Res. Dev. Cell Biol..

[48] Fahn A. (1979). Secretory Tissues in Plants.

[49] Fahn A. (2000). Structure and function of secretory cells. Adv. Bot. Res..

[50] Lewinsohn E., Dudai N., Tadmor Y., Katzir I., Ravid U., Putievsky E., Joel D.M.  (1998). Histochemical localization of citral accumulation in lemongrass leaves (*Cymbopogon citratus* (DC.) Stapf., Poaceae). Ann. Bot.-London.

[51] Gross M., Joel D.M., Cohen Y., Bar E., Friedman J., Lewinsohn E.  (2006). Ontogenesis of mericarps of bitter fennel (*Foeniculum vulgare* Mill. var. *vulgare*) as related to *t*-anethole accumulation. Isr. J. Plant Sci..

[52] Karcz J., Tomczok J.  (1987). Microstructural features of seeds surface in 6 species of the genus *Nigella* L. (Ranunculaceae). Acta Biol..

[53] Kanazawa K., Sakakibara H. (2000). High content of dopamine, a strong antioxidant, in Cavendish banana. J. Agric. Food Chem..

[54] Fait A., Angelovici R., Less H., Ohad I., Urbanczyk-Wochniak E., Fernie A.R., Galili G. (2006). Arabidopsis Seed development and germination is associated with temporally distinct metabolic switches. Plant Physiol..

[55] Hafez Y.M.  (2008). Effectiveness of the antifungal black seed oil against powdery mildews of cucumber (*Podosphaera xanthii*) and barley (*Blumeria graminis* f.sp. *hordei*). Acta Biol. Szeged..

[56] Maraka A., Al-Sharo’a N.F., Farah H., Elbjeirami W.M., Shakya A.K., Sallal A.K. (2007). Effect of *Nigella sativa* extract and oil on aflatoxin production by *Aspergillus flavus*. Turk. J. Biol..

[57] Chaubey M.K. (2007). Insecticidal activity of *Trachyspermum ammi* (Umbelliferae)*, Anethum graveolens* (Umbelliferae) and *Nigella sativa* (Ranunculaceae) essential oils against stored-product beetle *Tribolium castaneum* Herbst (Coleoptera: Tenebrionidae). Afr. J. Agric. Res..

[58] Dudai N., Larkov O., Putievsky E., Lerner H.R., Ravid U., Lewinsohn E., Mayer A.M. (2000). Biotransformation of constituents of essential oils by germinating wheat seed. Phytochemistry.

[59] Reynolds T.  (1987). Comparative effect of alicyclic compounds and quinones on inhibition of lettuce fruit germination. Ann. Bot.-London.

[60] Dudai N., Larkov O., Ravid U., Putievsky E., Lewinsohn E.  (2001). Developmental control of monoterpene content and composition in *Micromeria fruticosa* (L.) Druce. Ann. Bot.-London.

[61] Lisec J., Schauer N., Kopka J., Willmitzer L., Fernie A.R. (2006). Gas chromatography mass spectrometry-based metabolite profiling in plants. Nat. Protoc..

[62] Roessner U., Luedemann A., Brust D., Fiehn O., Linke T., Willmitzer L., Fernie A.R. (2001). Metabolic profiling allows comprehensive phenotyping of genetically or environmentally modified plant systems. Plant Cell.

[63] Luedemann A., Strassburg K., Erban A., Kopka J. (2008). TagFinder for the quantitative analysis of gas chromatography-Mass spectrometry (GC-MS)-based metabolite profiling experiments. Bioinformatics.

[64] Erban A., Schauer N., Fernie A.R., Kopka J. (2007). Nonsupervised construction and application of mass spectral and retention time index libraries from time-of-flight gas chromatography-mass spectrometry metabolite profiles. Method. Mol. Cell. Biol..

[65] Hummel J., Selbig J., Walther D., Kopka J., Nielsen J., Jewett M. (2007). The Golm Metabolome Database: A Database for GC-MS Based Metabolite Profiling. Metabolomics.

[66] Schauer N., Semel Y., Roessner U., Gur A., Balbo I., Carrari F., Pleban T., Perez-Melis A., Bruedigam C., Kopka J. (2006). Comprehensive metabolic profiling and phenotyping of interspecific introgression lines for tomato improvement. Nat. Biotechnol..

[67] Brundrett M.C., Kendrick B., Peterson C.A. (1991). Efficient lipid staining in plant material with sudan red 7B or fluorol yellow 088 in polyethylene glycol-glycerol. Biotech. Histochem..

[68] Caissard J.C., Bergougnoux V., Martin M., Mauriat M., Baudino S.  (2006). Chemical and histochemical analysis of ‘Quatre Saisons Blanc Mousseux’, a Moss Rose of the *Rosa* X *damascena* Group. Ann. Bot.-London.

